# Few-Shot Fault Diagnosis of Railway Switch Machines Using Regularized Supervised Contrastive Meta-Learning

**DOI:** 10.3390/s26092827

**Published:** 2026-05-01

**Authors:** Shanrong Li, Qingsheng Feng, Zhun Han, Shuai Xiao, Zhi Tao, Yafei Wang, Yiyang Zou, Hong Li

**Affiliations:** 1 School of Electrical Engineering, Dalian Jiaotong University, Dalian 116028, China; shanr2024@126.com (S.L.); fqs@djtu.edu.cn (Q.F.); 13604228286@163.com (Z.H.); 13064042850@163.com (Z.T.); wangyf66789@126.com (Y.W.); 18840838965@163.com (Y.Z.); 2 Baotou Electric Power Section of China Railway Hohhot Bureau Group Co., Ltd., Baotou 014040, China; xiaos1002@126.com; 3 School of Railway Intelligent Engineering, Dalian Jiaotong University, Dalian 116028, China

**Keywords:** switch machine, fault diagnosis, few-shot, meta-learning, vibration signals

## Abstract

Railway switch machines are key devices in railway signal systems and have a critical impact on train operation safety. However, in real operating conditions, fault samples are scarce because field data collection is cumbersome and often constrained by safety requirements, which limits the diagnostic accuracy and generalization capability of traditional fault diagnosis methods in few-shot scenarios. To address the challenge posed by insufficient accuracy in railway switch machine state recognition using sensors under few-shot conditions, we propose a regularized supervised contrastive meta-learning (RSCML) fault diagnosis method for switch machines. First, the tri-axial vibration signals acquired from the throwing rod and the reducer are transformed into axis-wise STFT spectrograms and organized as a unified three-channel time-frequency representation for subsequent cross-channel feature learning. Second, channel expansion and attention enhancement are employed to obtain more informative feature representations among similar fault types under limited samples. Finally, the feature extractor is integrated into the regularized supervised contrastive ANIL framework, while multi-loss optimization and stability regularization jointly constrain the meta-learning training process. Experimental results show that the proposed method achieves a maximum accuracy of 99.73% on 3-way and 5-way few-shot tasks, together with an F1-score of up to 99.72%. In the cross-category generalization experiment, it achieves a 93.08% accuracy and a 92.84% F1-score, indicating improved robustness when the fault categories at test time differ from those used during meta-training. The proposed method shows superior classification performance and stronger generalization to unseen fault categories under the current dataset setting, which suggests promising potential for switch machine fault diagnosis under limited sample conditions.

## 1. Introduction

Switch machines are core devices in railway signal systems, and their reliability is directly related to train operation safety. Faults in switch machines may cause train delays and can even lead to serious safety incidents [1,2]. Therefore, conducting fault diagnosis for switch machines is of great practical significance. However, in real operating environments, it is difficult to acquire sufficient labelled fault data from switch machines, and the available fault data are extremely limited. As a result, conventional fault diagnosis methods that rely on large-scale training data often struggle in data-scarce scenarios [3,4]. In contrast, few-shot learning can rapidly capture fault characteristics from limited samples, enabling efficient and accurate fault diagnosis. Consequently, how to achieve effective fault diagnosis of switch machines under few-shot conditions has become a current research focus.

In recent years, extensive research on switch machine fault diagnosis has been conducted, with a large body of work developed within machine learning (ML) frameworks. ML methods typically rely on manually extracting features from sound signals, current signals, and other monitoring data, and then feeding these features into conventional machine learning models to automatically identify different fault types. For example, Li et al. [5] extracted features from the sound signals of switch machines using empirical mode decomposition (EMD), performed feature selection and dimensionality reduction via the ReliefF algorithm, and employed a support vector machine (SVM) for fault diagnosis. Lao et al. [6] proposed a fault diagnosis method for three-phase currents during normal-to-reverse and reverse-to-normal switching processes of switch machines by integrating multi-scale permutation entropy (MPE) with an improved LightGBM classifier. Wang et al. [7] developed a diagnosis approach for switch machine power signals that combines segmentalized minimum-redundancy–maximum-relevance (mRMR) feature selection with a cost-sensitive extreme learning machine (ELM) under fixed input conditions. Song et al. [8] applied variational mode decomposition (VMD) to preprocess power signals and subsequently adopted a kernel fuzzy C-means clustering algorithm to distinguish different fault types. Although the above methods have achieved promising diagnostic performance, they still heavily depend on hand-crafted features [9] and require extensive manual effort for feature extraction and a high level of domain expertise [10]. Consequently, when operating conditions are complex or non-stationary, it becomes difficult for these approaches to fully capture the latent characteristics and time-frequency evolution patterns of input signals [11].

To overcome the heavy dependence of conventional ML methods on hand-crafted features and domain expertise, deep learning (DL) methods have been increasingly applied to fault diagnosis of switch machines. By constructing multi-layer neural networks, DL enables end-to-end feature extraction and classification, allowing complex spatiotemporal patterns in input signals to be automatically learned. For instance, Chen et al. [12] converted one-dimensional current signals into two-dimensional images and then fed them into a convolutional neural network (CNN), thereby transforming the traditional time-series problem into an image classification task. Xiao et al. [13] specifically addressed the issue of data imbalance under dual-machine traction conditions for railway switch machines by proposing a fault diagnosis model based on a deep feature fusion network, which effectively improves fault recognition accuracy and robustness under complex operating conditions. Chen et al. [14] focused on algorithmic design and proposed a switch machine fault diagnosis method that integrates multi-head channel self-attention and residual connections into a deep convolutional neural network, enabling adaptive extraction of salient features in complex signal environments. Cao et al. [15] proposed a switch machine fault diagnosis approach based on deep random forest fusion, which combines deep feature extraction with a random forest classifier to achieve the fusion of tri-axial vibration signals. However, these methods typically require large quantities of high-quality labeled data to ensure diagnostic accuracy [16]. In real industrial scenarios, especially for critical equipment such as switch machines, fault samples are often scarce [17], and certain fault types are difficult to reproduce, leading to severe data insufficiency [18]. The inherent scarcity of labeled fault data in field operation markedly limits the deployment and practical applicability of switch machine fault diagnosis methods based on DL.

Few-shot learning methods have emerged as an effective approach to address the above challenges. FSL aims to learn a model that can accurately classify query samples when only a limited number of labeled examples are available [19]. Its core idea is to enhance the model’s generalization ability by fully exploiting prior knowledge [20], sharing a common feature space [21], or adopting meta-learning strategies [22], so that satisfactory diagnostic accuracy can still be achieved under data-scarce conditions. As an emerging paradigm within few-shot learning, meta-learning plays an important role in alleviating data scarcity by enabling effective model adaptation from limited labeled data. Wu et al. [23] proposed a few-shot transfer learning method that leverages meta-learning to handle variable working conditions and scarce fault samples in machine fault diagnosis. Yang et al. [24] developed a cross-domain fault diagnosis method based on model-agnostic meta-learning (MAML) to enhance adaptation to changing operating conditions when only limited fault data are available. Lao et al. [25] proposed a semi-supervised weighted prototypical network that leverages unlabeled data to refine prototypes and improve few-shot fault diagnosis of turnout switch machines. These studies indicate that meta-learning can be effective in data-scarce scenarios, and its performance still depends strongly on whether the input signals are transformed into a representation that exposes fault-sensitive structures shared across tasks. Therefore, effectively mapping one-dimensional time-series signals into a representation space that can adequately capture their time-frequency characteristics has become a key challenge in applying few-shot learning to switch machine fault diagnosis.

To address the above issues, this paper proposes a regularized supervised contrastive meta-learning (RSCML) fault diagnosis method for switch machines. First, tri-axial vibration signals (X, Y, and Z), which are collected from on-site railway switch machines, are transformed into time-frequency representations via short-time Fourier transform (STFT) and fused along the channel dimension to construct three-channel time-frequency images in order to integrate complementary information across axes and obtain a more informative and robust representation for fault diagnosis. Subsequently, an attention-enhanced feature extraction module is built by incorporating a squeeze-and-excitation (SE) channel attention mechanism and a spatial attention mechanism, which adaptively strengthen informative channel-wise features and highlight most distinctive fault feature areas, thus enhancing the separability of fault features under few-shot conditions. Finally, the feature extraction network is embedded into a regularized supervised contrastive ANIL meta-learning framework, in which only the task-specific classification layer is updated while the shared feature extraction layers are kept fixed. This design reduces computational overhead, improves the stability of feature transfer, and enables efficient fault recognition in multi-task few-shot training. And in practical railway maintenance scenarios, the proposed method can serve as a data-driven decision-support tool for switch machine condition monitoring under limited labeled data. For example, it can be applied to newly deployed or recently overhauled switch machines and to rare fault categories encountered in field operation, thereby assisting maintenance personnel in fault identification and inspection prioritization. The major contributions of this paper can be summarized as follows:

(1) The tri-axial vibration signals of the switch machine are transformed into time-frequency spectrograms via STFT and fused into three-channel images in order to enhance the representational capacity of state-related features so that the model can capture key fault-sensitive characteristics and improve fault recognition performance.
(2) An attention-enhanced module that integrates SE channel attention and spatial attention is designed, enabling the model to adaptively emphasize key channels and salient time-frequency regions, thus strengthening the discriminative power of the learned feature representations.
(3) A multi-loss regularization strategy is proposed within the ANIL meta-learning scheme to establish the RSCML, which effectively improves feature separability by enhancing intra-class compactness and inter-class separation, thereby significantly improving diagnostic accuracy under few-shot conditions.

The remainder of this paper is organized as follows: Section 2 the selection of the input signals and the theoretical knowledge of the methods used in this paper. Section 3 describes the proposed fault diagnosis model in detail. Section 4 introduces the data acquisition process and experimental settings. Section 5 reports and discusses the experimental results. Lastly, Section 6 concludes this work and outlines directions for future research.

## 2. Methodology

### 2.1. Vibration Signal Selection

Vibration signals provide a rich information description of the dynamic response of switch machines during turnout switching operations. Mechanical faults such as jamming, loosening, or abnormal friction are often manifested as changes in impact intensity and time-frequency energy distribution [26]. In many cases, these changes are often reflected earlier and more explicitly in vibration responses than in other signals such as current and power signals. Therefore, vibration signals constitute a sensitive and suitable data variable for supporting fault diagnosis.

In this study, tri-axial vibration data are acquired in a realistic field environment using two sensors (WTVB01-485; WitMotion Shenzhen Co., Ltd., Shenzhen, China) mounted on the throwing rod and the reducer of a ZD6 switch machine. Signals along the X, Y, and Z directions are sampled at 1 kHz, and an 8 s window to cover a complete switching operation. Since switch machine vibration is inherently non-stationary, discriminative patterns are frequently embedded in time-frequency structures rather than raw time-domain waveforms, which motivates subsequent time-frequency analysis.

### 2.2. Principle of Short-Time Fourier Transform

In the field of signal analysis, the Fourier transform (FT) is a classical frequency-domain analysis tool that decomposes a time-domain signal into a series of sinusoidal components at different frequencies, thereby revealing its spectral characteristics. However, the Fourier transform implicitly assumes that the signal remains stationary over the entire observation interval. This assumption often fails when dealing with non-stationary signals such as mechanical vibration signals from machines. To address this limitation, the short-time Fourier transform (STFT) [27] was introduced to provide a joint time-frequency representation of signals. By capturing how spectral components evolve over time, STFT is particularly suitable for analyzing non-stationary vibration signals encountered in industrial data acquisition scenarios.

The basic principle of STFT is to apply a finite-length window that slides along the signal, under the assumption that the signal within each windowed segment can be regarded as locally stationary. A Fourier transform is then performed on each windowed segment, allowing the local spectral content at different time instants to be obtained. In this manner, the signal can be simultaneously characterized in both the time and frequency domains [28].

Specifically, let $x\left(t\right)$ denote a one-dimensional continuous signal and $w\left(t\right)$ denote a window function; the STFT can then be defined as follows:

(1) $$X\left(\tau ,\omega \right)=\int_{-\infty }^{+\infty }x(t)w\left(t-\tau \right)e^{-j\omega t}dt$$

where τ is the time-shift parameter of the window function and ω is the angular frequency.

**Figure 1:**
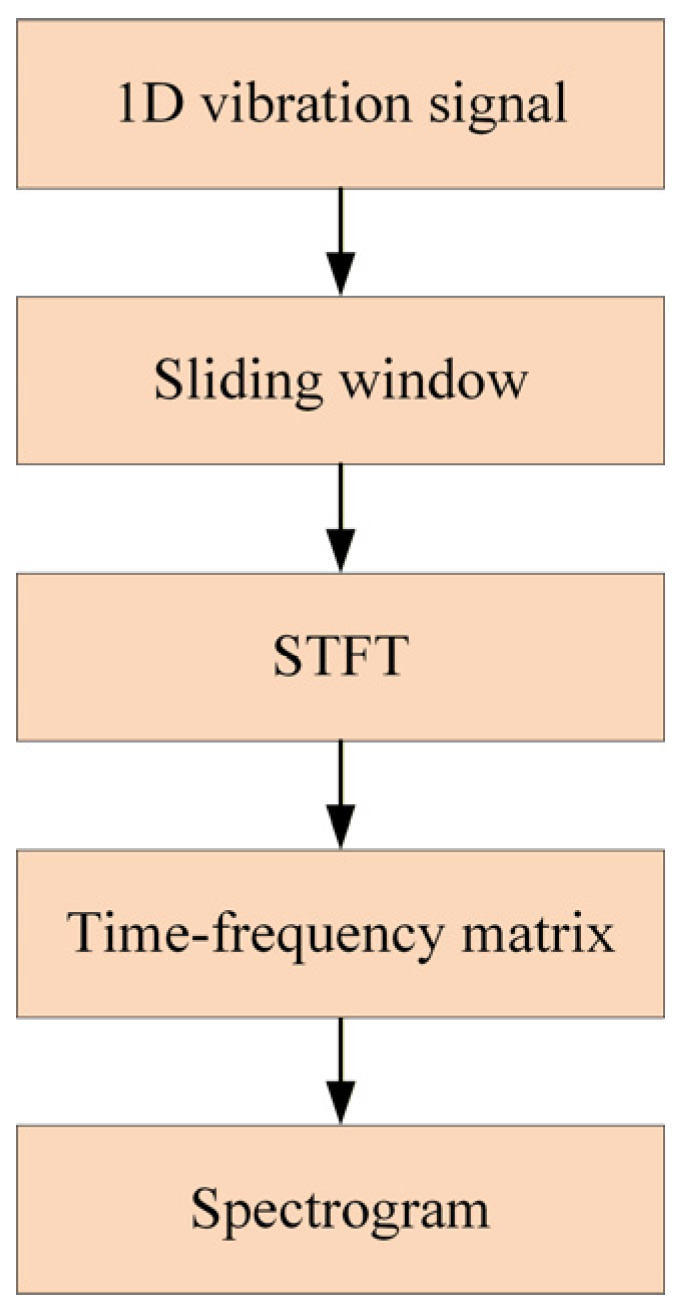
Flowchart of the STFT-based process from a one-dimensional vibration signal to a time-frequency spectrogram.

After applying the short-time Fourier transform (STFT), a two-dimensional matrix is obtained, where the horizontal axis corresponds to time, the vertical axis corresponds to frequency, and each element represents the signal magnitude at a specific time-frequency point. The matrix is converted to a logarithmic scale to compress the dynamic range and normalized to a fixed intensity range. The normalized values are then mapped to pixel intensities using a predefined colormap and rendered as a time-frequency spectrogram, with time and frequency serving as the horizontal and vertical coordinates, respectively. For clarity, the STFT-based transformation from the original one-dimensional vibration signal to the final spectrogram is illustrated in Figure 1.

Such a spectrogram not only preserves the information of both the time and frequency domains, but also intuitively reflects the evolution of the signal spectrum over time [28]. In this work, STFT serves as the basis for converting one-dimensional tri-axial vibration signals into time-frequency images for subsequent feature extraction.

In this work, STFT was adopted as a practical and unified time-frequency representation for vibration signal preprocessing. Railway switch machine vibration signals are non-stationary and may contain transient fault-related components, for which time-frequency analysis is a reasonable preprocessing strategy [29]. STFT provides a joint description in the time and frequency domains, and STFT-based spectrograms have been widely used as image inputs in fault diagnosis studies [30,31]. Since the focus of this study is the proposed diagnosis framework rather than a dedicated benchmark of different time-frequency transforms, STFT was used as a practical preprocessing method throughout all experiments.

### 2.3. Attention Mechanisms

Attention mechanisms are inspired by the selective attention of the human visual system. They assign higher weights to informative and task-relevant components of the input, guiding the model to learn more discriminative feature representations [32].

According to the dimension on which they operate, attention mechanisms can be broadly divided into channel attention and spatial attention. Channel attention focuses on the relative importance of different feature channels and adaptively re-weights them to emphasize key feature maps. A typical channel-attention structure is the squeeze-and-excitation (SE) module proposed by Hu et al. [33], which performs global average pooling followed by a nonlinear transformation to generate channel-wise weights and thus achieves adaptive channel recalibration. Specifically, given a feature map $U\in {\mathbb{R}}^{C\times H\times W}$, the calculation process of squeeze can be defined as follows:

(2) $$z_{c}=\frac{1}{HW}\sum_{i=1}^{H}\sum_{j=1}^{W}U_{c}\left(i,j\right),\;c=1,\ldots ,C$$

where H and W are the height and width of the feature map, respectively, and C is the number of channels.

**Figure 2:**
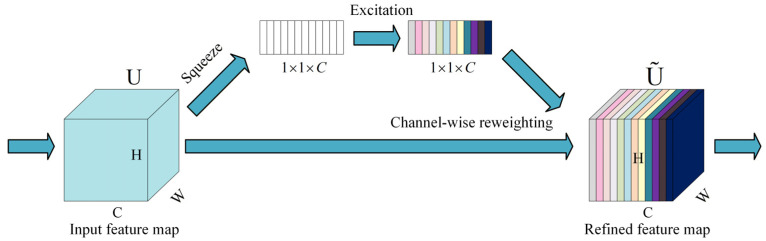
Schematic illustration of the channel attention mechanism based on squeeze-and-excitation.

Then the excitation operation produces channel-wise weights, which can be denoted as follows:

(3) $$s=\sigma \left(W_{2}\;\delta \left(W_{1}z\right)\right)$$

where δ(⋅) and σ(⋅) denote ReLU and Sigmoid functions, respectively, and $W_{1}$, $W_{2}$ are learnable parameters. For clarity, a schematic illustration of the channel attention mechanism is provided in Figure 2.

**Figure 3:**
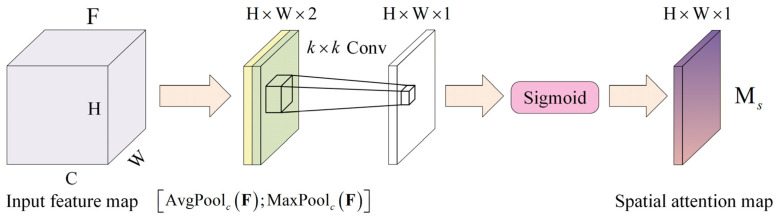
Schematic illustration of the spatial attention mechanism.

Spatial attention (SA) emphasizes the spatial distribution of features: by learning the correlation among different spatial locations, it assigns higher weights to salient regions in the feature map and suppresses less informative areas [34]. A common formulation computes a spatial attention map by aggregating channel-wise statistics and applying a convolutional operator, and then re-weights the original feature map accordingly, which can be denoted as follows:

(4) $$M_{s}=\sigma \left(f^{k\times k}\left(\left[{\mathrm{AvgPool}}_{c}\left(F\right);{\mathrm{MaxPool}}_{c}\left(F\right)\right]\right)\right)$$

where $\left[\;\cdot \;;\;\cdot \right]$ denotes channel-wise concatenation, $f^{k\times k}$ is a convolution with kernel size k×k, and $F\in {\mathbb{R}}^{C\times H\times W}$ is a feature map. For clarity, a schematic illustration of the spatial attention mechanism is provided in Figure 3.

And then the weighting process of the feature maps can be denoted as

(5) $$\tilde{F}=M_{s}⊙F$$

where ⊙ denotes element-wise multiplication with broadcasting.

In the proposed model, these two types of attention mechanisms are jointly exploited. Channel attention is used to identify and enhance informative vibration-related channels, while spatial attention encourages the network to focus on time-frequency regions that are most sensitive to fault patterns, forming the basis of the attention-enhanced feature extraction module introduced later.

### 2.4. Meta-Learning

Meta-learning is a class of algorithms that enable a model to rapidly adapt to new tasks using only a small amount of data [35]. The key idea is to allow the model to acquire, through experience over a distribution of tasks, a general capability of “how to learn,” so that it can quickly adjust its parameters and achieve fast convergence under data-scarce conditions commonly encountered in field data acquisition. In few-shot fault diagnosis, this property is particularly important because only a limited number of labeled samples are usually available for each fault category, while reliable adaptation to new diagnostic tasks is still required.

The basic framework of meta-learning typically consists of two levels: an outer meta level, which is responsible for learning a global update strategy or parameter initialization; and an inner task level, which performs rapid learning and fine-tuning for each specific task [36]. Through this mechanism, the model is optimized not only for performance on a single training task, but also for its ability to adapt quickly across related tasks. As a result, meta-learning is well suited to few-shot diagnosis scenarios in which the support set is small and efficient adaptation is necessary.

Among various meta-learning algorithms, approaches based on optimization are widely adopted. A representative example is the model-agnostic meta-learning (MAML) algorithm proposed by Finn et al. [37], which learns a set of model initialization parameters over a task distribution such that good performance on a new task can be achieved with only a few gradient updates. Compared with metric-based meta-learning methods, optimization-based approaches are more suitable for scenarios where task distributions exhibit substantial variations in signal characteristics, as they directly optimize task-specific performance based on gradient adaptation.

Specifically, let θ denote the model parameters and $\left.\left\{T_{i}\right.\right\}$ denote the set of tasks, where each task $T_{i}$ is associated with a support set $D_{i}^{train}$ and a query set $D_{i}^{test}$. In the inner loop, MAML performs task-specific adaptation by updating θ using the loss computed on the support set. After a complete parameter update, the adapted parameters for task $T_{i}$ are obtained, which are denoted as:

(6) $$\theta_{i}^{\prime }=\theta -\alpha \nabla_{\theta }L_{T_{i}}\left(f_{\theta },D_{i}^{train}\right)$$

where α is the inner-loop learning rate, $f_{\theta }$ is the model parameterized by θ, and $L_{T_{i}}$ denotes the task loss.

In the outer loop, the shared parameters θ are optimized to improve generalization across tasks. Specifically, after obtaining the task-adapted parameters $\theta_{i}^{\prime }$ in the inner loop, the adapted model $f_{\theta_{i}^{\prime }}$ is evaluated on the query set $D_{i}^{test}$. The meta-objective is constructed by aggregating the query losses over a batch of tasks, and the meta-update is defined as follows:

(7) $$\theta \leftarrow \theta -\beta \nabla_{\theta }\sum_{T_{i}}L_{T_{i}}\left(f_{\theta_{i}^{\prime }},D_{i}^{test}\right)$$

where β is the outer-loop learning rate.

**Figure 4:**
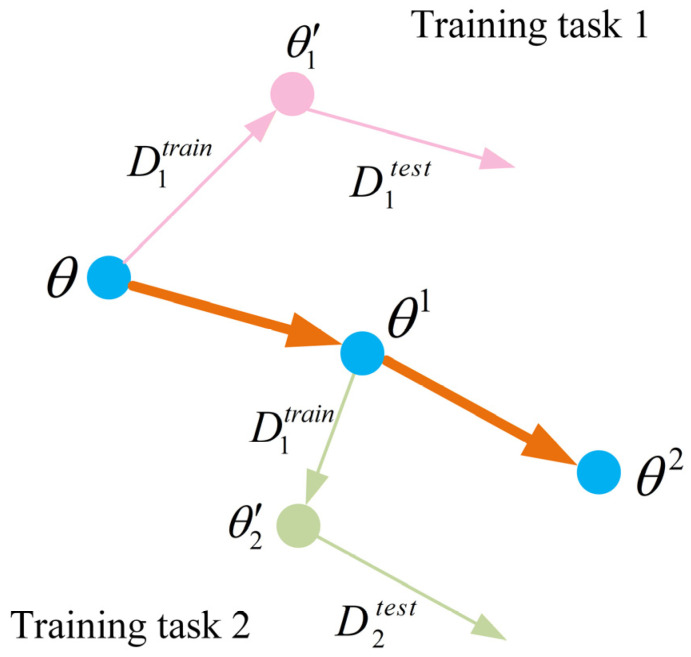
Schematic illustration of the MAML algorithm.

Through iterative optimization of (6) and (7), the model learns a set of initialization parameters with good transferability, enabling rapid convergence under few-shot conditions. When the meta-update is performed using only a single task, the resulting procedure is illustrated in Figure 4.

As shown in Figure 4, for each meta-training task $T_{i}$, the model parameters are adapted from the shared initialization θ to task-specific parameters $\theta_{i}^{\prime }$ using the support set $D_{i}^{train}$, and the meta-loss is computed on the query set $D_{i}^{test}$. The shared parameters θ are then updated by minimizing the aggregated query losses over tasks, producing a new initialization $\theta^{i}$ that enables rapid adaptation.

In the field of industrial equipment fault diagnosis, particularly for switch machines, the operating conditions are highly complex and variable, such that even the same fault type may exhibit substantially different characteristics under different working states. Consequently, compared with pursuing cross-task generalization in a broad sense, the primary motivation for employing meta-learning strategies in this domain is to enhance the model’s ability to rapidly adapt to tasks of the same category, thereby addressing fault diagnosis problems under few-shot conditions.

## 3. The Proposed Method

**Figure 5:**
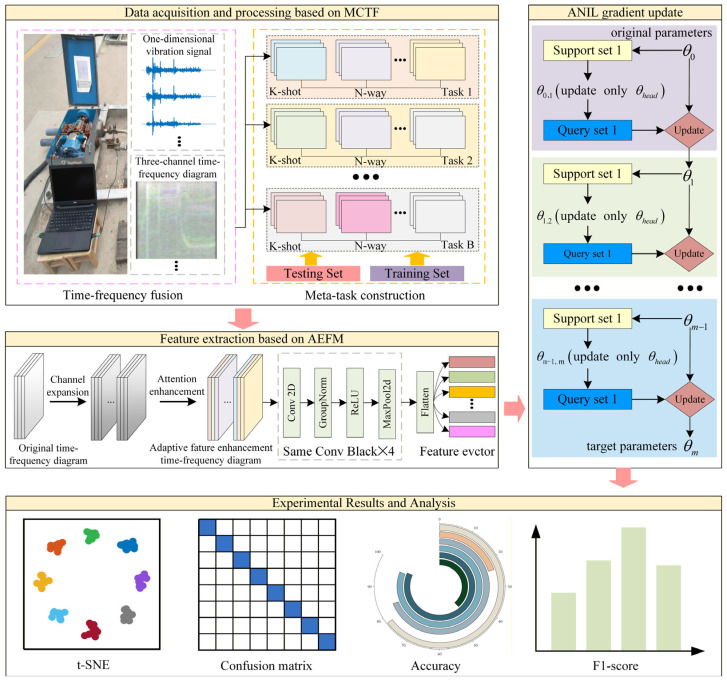
Fault diagnosis framework of the proposed RSCML method.

To address the challenges of limited fault samples and complex operating condition variations in switch machine fault diagnosis, this paper develops a regularized supervised contrastive meta-learning model. As shown in Figure 5, the proposed method first performs multi-dimensional time-frequency feature fusion via STFT, then strengthens the representation of critical information through channel expansion and an attention fusion mechanism, and finally integrates the regularized supervised contrastive meta-learning framework based on ANIL to enable rapid learning and accurate fault diagnosis under few-shot conditions. The model is described in detail as follows.

### 3.1. Multi-Channel Time-Frequency Feature Fusion

During the operation of a switch machine, vibration signals generated by components such as the throwing rod and the reducer often exhibit pronounced non-stationary characteristics. Vibration responses along different directions often encode distinct dynamic behaviors and fault-related signatures; therefore, analyzing the vibration signal from only a single axis may ignore the coupling among multi-directional features. Moreover, for one-dimensional vibration signals, certain fault patterns can manifest much more distinctly when observed jointly in the time and frequency domains. Consequently, relying on features from only one domain may fail to provide sufficiently informative representations for reliable diagnosis under complex operating conditions.

**Figure 6:**
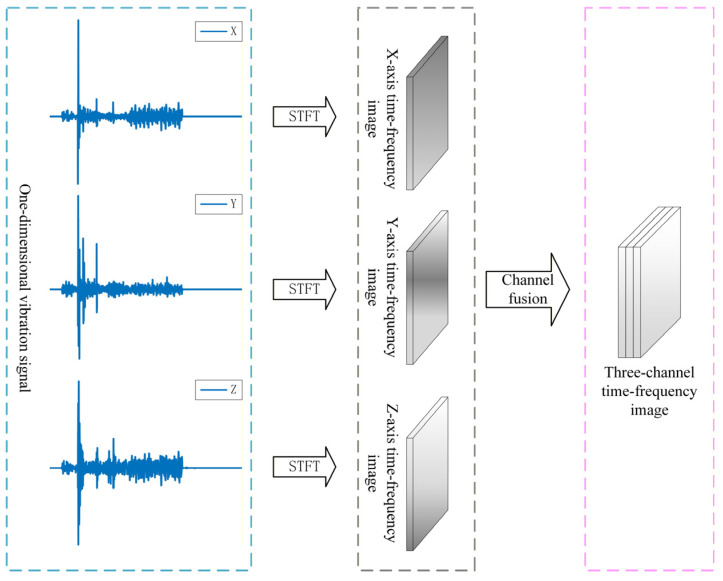
Multi-channel time-frequency fusion.

To this end, we propose a multi-channel time-frequency fusion module (MCTF). As shown in Figure 6, the time-domain vibration signals are first transformed into the time-frequency domain via short-time Fourier transform (STFT) to obtain joint time-frequency representations. For the vibration signal $x_{k}\left(t\right)$, its STFT can be formulated as follows:

(8) $$S_{k}\left(t,f\right)=\int_{-\infty }^{+\infty }x_{k}\left(\tau \right)w\left(t-\tau \right)e^{-j2\pi f\tau }d\tau$$

where $w\left(t\right)$ is the window function.

By applying the sliding-window transform segment by segment, the local spectral distribution of the signal at different time instants can be obtained. The resulting time-frequency spectrum is then converted into a two-dimensional time-frequency energy matrix through magnitude computation and normalization. Subsequently, the time-frequency spectra of the three axes are stacked along the channel dimension to form a three-channel time-frequency image:

(9) $$I\left(t,f\right)=\left[S_{x}\left(t,f\right),S_{y}\left(t,f\right),S_{z}\left(t,f\right)\right]$$

where I(t,f) denotes the constructed three-channel time-frequency image at time t and frequency f. $S_{x}\left(t,f\right)$, $S_{y}\left(t,f\right)$, and $S_{z}\left(t,f\right)$ are the corresponding normalized time-frequency spectra obtained from the STFT of the vibration signals.

In addition, to ensure consistency and comparability of the input images, all time-frequency spectrograms are subjected to normalization and size resampling, so that samples share the same spatial resolution. After this step, the original one-dimensional vibration signals of the switch machine are converted into three-channel images of size 128 × 128 by stacking the synchronized axis-wise spectrograms into a unified representation. The stacked three-channel representation preserves complementary information from different vibration axes, while subsequent feature extraction through the channel-expansion convolution and attention modules enables cross-channel feature learning from the stacked multi-axis representation.

### 3.2. Channel Expansion and Attention Enhancement

Under few-shot conditions, extracting highly discriminative representations from limited samples is crucial for accurate diagnosis. To this end, we propose an attention-enhanced feature module (AEFM) based on CNN components. AEFM first increases feature dimensionality through channel expansion, and then employs SE channel attention together with spatial attention to adaptively select and amplify informative patterns. The refined representations are subsequently fed into multi-layer convolutional blocks for hierarchical feature extraction.

**Figure 7:**
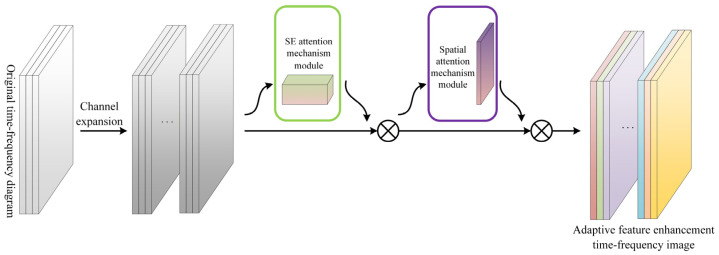
Channel expansion and attention enhancement in the proposed AEFM.

As shown in Figure 7 and 3 × 3 convolution is first applied to the input three-channel time-frequency image to perform channel expansion, and a sigmoid activation function is then used to produce an initial feature map, which is expressed as follows:

(10) $$F_{0}=f_{3\times 3}\pi \left(I\right)=W_{3\times 3}*I+b$$

where I denotes the input image, $W_{3\times 3}$ and b represent the convolution kernel and bias, * denotes the convolution operation, and $F_{0}\in {\mathbb{R}}^{C^{\prime }\times H\times W}$ is the resulting feature map.

Next, an SE channel attention mechanism is introduced. Specifically, global average pooling is first applied to compress the feature map. Then two fully connected layers are used to implement a nonlinear transformation, and a sigmoid function generates the channel-wise attention weights. Finally, the feature map is reweighted channel-wise as follows:

(11) $$z_{c}=\frac{1}{HW}\sum_{i=1}^{H}\sum_{j=1}^{W}F_{0}^{c}\left(i,j\right)$$

where H and W denote the height and width of the feature map, respectively; $F_{0}^{c}\left(i,j\right)$ denotes the value at the spatial location $\left(i,j\right)$ of the c-th channel.

(12) $$s_{c}=\sigma \left(W_{2}\;\delta \left(W_{1}z_{c}\right)\right)$$

where δ(⋅) denotes the ReLU activation function; σ(⋅) denotes the sigmoid activation function. $W_{1}$ and $W_{2}$ are the weight matrices of the first and second fully connected layers, respectively.

(13) $$F_{c}=s_{c}⊗F_{0}$$

where $F_{c}$ represents the modulated features after channel-wise recalibration; and ⊗ represents channel-wise multiplication with broadcasting.

To further enhance the model’s sensitivity to features at different spatial locations, a spatial attention mechanism is introduced. It computes an integrated weight for each pixel location, guiding the model to focus on regions with concentrated energy and salient variations, which can be denoted as

(14) $$M_{s}\left(F_{c}\right)=\sigma \left(f^{7\times 7}\left(\left[\mathrm{AvgPool}\left(F_{c}\right);\mathrm{MaxPool}\left(F_{c}\right)\right]\right)\right)$$

where $M_{s}(\cdot )$ denotes the spatial attention function, and $\mathrm{AvgPool}\left(F_{c}\right)$ and $\mathrm{MaxPool}\left(F_{c}\right)$ represent average pooling and max pooling applied to the feature map $F_{c}$ respectively; $\left[\;\cdot \;;\;\cdot \right]$ denotes the concatenation operation; $f^{7\times 7}$ denotes a 7 × 7 convolution.

(15) $$F_{s}=M_{s}\left(F_{c}\right)⊙F_{c}$$

where $F_{s}$ is the weighted feature map obtained after applying both channel attention and spatial attention; ⊙ denotes element-wise multiplication with broadcasting.

Lastly, $F_{s}$ is fed into a multi-layer convolutional module composed of four identical convolutional blocks to extract deeper representations. With this architecture, the network further strengthens fault-relevant time-frequency patterns, yielding more discriminative feature representations

Through the hierarchical integration of channel expansion, channel attention, and spatial attention, we construct AEFM, which not only enlarges the feature dimensionality but also produces high-dimensional representations that are flattened and fed into a linear classifier. This design facilitates rapid discrimination among different fault categories within the meta-learning framework.

### 3.3. Regularized Supervised Contrastive Meta-Learning Framework with ANIL

Conventional deep learning models typically rely on large-scale data to perform end-to-end optimization; when training data are insufficient, overfitting and unstable training are likely to occur. To alleviate this issue, we construct a regularized supervised contrastive meta-learning framework based on ANIL [38]. This framework performs optimization at the task level, enabling the model to rapidly adapt to similar fault diagnosis tasks with only a few samples. Compared with MAML, ANIL updates only the classification head for each task while keeping the feature extractor fixed. This strategy markedly reduces the computational cost of backpropagation and improves training stability and convergence under limited sample conditions.

In the field of meta-learning, the core objective is to learn a model $f_{\theta }$ that can rapidly adapt to a variety of tasks. In few-shot classification, tasks are typically formulated as an *N*-way and *K*-shot problem, where *N* denotes the number of classes in a task and *K* denotes the number of samples per class. Assume that the fault task distribution is $p\left(T\right)$, from which *B* tasks are sampled. For each task $T_{i}$, a support set $D_{i}^{s}=\left\{x_{s},y_{s}\right\}$ containing *N* × *K* samples and a query set $D_{i}^{q}=\left\{x_{q},y_{q}\right\}$ containing *N* × *M* samples are constructed, and the samples in the support and query sets are strictly nonoverlapping. Then the meta parameters are initialized as θ. Using the support set, the model performs k gradient updates in the inner loop. During this process, only the task specific classifier parameters are updated, while the shared feature extractor parameters θ remain fixed. Let j denote the inner-loop iteration, where j range from 0 to k−1. The corresponding update rule is denoted as

(16) $$\theta_{i,j+1}=\theta_{i,j}-\alpha \nabla_{\theta }\sum_{i=1}^{B}\frac{1}{\left|D^{s}\right|}\sum_{\left(x_{s},y_{s}\right)\in D^{s}}L\left(f_{\theta_{i,k}}\left(x_{s}\right),y_{s}\right)$$

where α is the inner-loop learning rate, and $f_{\theta_{i,k}}\left(x_{s}\right)$ denotes the feature representation extracted from the support set, $\left|\cdot \right|$ consistently represents count of elements when applied to a set.

After completing the inner-loop updates for all tasks and obtaining the updated parameters, the meta model parameters are optimized by minimizing the average query loss over the B tasks. To enhance the discriminability of learned representations, we introduce a joint multi-loss optimization strategy within the ANIL framework. Specifically, the cross-entropy loss and the supervised contrastive loss (SupCon) [39] are jointly used to constrain model training. The cross-entropy loss maximizes the log likelihood of the correct class, ensuring that the classifier can quickly learn the decision boundary among different fault categories, which can be expressed as

(17) $$L_{CE}=\sum_{i=1}^{B}\frac{1}{\left|D^{q}\right|}\sum_{\left(x_{q},y_{q}\right)\in D^{q}}L\left(f_{\theta_{i,k}}\left(x_{q}\right),y_{q}\right)$$

where $f_{\theta_{i,k}}\left(x_{q}\right)$ denotes the feature representation of the query set.

However, relying solely on the cross-entropy loss often fails to yield well-clustered features under few-shot conditions. Therefore, we incorporate SupCon, which uses label information to treat samples from the same class as positive pairs, thereby promoting intra-class compactness and inter-class separability in the feature space [40].

Assume that the number of samples in the current batch is N, with feature vector $z_{i}$ and labels $y_{i}$. Then, the set of positive samples for sample i is defined as

(18) $$P\left(i\right)=\left\{j|j\neq i,y_{j}=y_{i}\right\}$$

Accordingly, the supervised contrastive loss can be written as

(19) $$L_{\sup}=\sum_{i=1}^{N}\frac{1}{\left|P(i)\right|}\sum_{p\in P(i)}-\log\frac{\exp\left(sim\left(z_{i},z_{p}\right)/\tau \right)}{\sum_{a=1,a\neq i}^{N}\exp\left(sim\left(z_{i},z_{a}\right)/\tau \right)}$$

where $sim\left(\cdot \right)$ denotes cosine similarity and τ is the temperature parameter. Compared with using cross-entropy loss alone, supervised contrastive learning loss makes fuller use of label information and encourages a more stable and more discriminative feature structure in few-shot fault recognition.

By combining the two losses above, the overall loss of RSCML is formulated as follows:

(20) $$L_{RSC\mathrm{ML},i}=L_{CE,i}+\lambda L_{\sup,i}$$

where λ is the weighting coefficient for the contrastive term, which is used to balance the relative contributions of cross-entropy classification loss and supervised contrastive loss.

In addition, to mitigate overfitting that may arise from the enriched feature space, a stability regularization (SR) strategy is introduced. It combines Dropout and L2 regularization, so that the model can converge efficiently while maintaining stable classification performance when recognizing the same fault type under different operating conditions.

Specifically, Dropout regularization reduces the model’s dependence on local features by randomly discarding a subset of neuron connections [41]:

(21) $${\tilde{h}}_{i}=r_{i}\cdot h_{i},\;r_{i}\sim \mathrm{Bernoulli}(p)$$

where p denotes the retention probability. And L2 regularization improves stability by adding a weight penalty term to the loss function, which encourages smoother parameter distributions [42].

By minimizing the average RSCML loss on the query sets after task adaptation, the model learned representation to support rapid classifier adjustment and robust generalization across tasks, which is defined as

(22) $$L_{RSC\mathrm{ML}}=\frac{1}{B}\sum_{i=1}^{B}L_{RSC\mathrm{ML},i}$$

The final parameter update of the model is as follows:

(23) $$\theta \leftarrow \theta -\beta \nabla_{\theta }L_{RSC\mathrm{ML}}$$

where β is the outer-loop learning rate.

### 3.4. The Process of Fault Diagnosis

To address scarce on-site fault samples for railway switch machines, we propose a regularized supervised contrastive meta-learning method with multi-channel time-frequency fusion and attention enhancement. This advanced meta-learning paradigm for vibration data analysis can efficiently extract fault features from limited vibration signals.

Algorithm 1 summarizes the proposed regularized supervised contrastive meta-learning framework.**Algorithm 1** Regularized Supervised Contrastive ANIL with Multi-Channel Time-Frequency Fusion and Attention Enhancement**Require**: task distribution $p\left(T\right)$, vibration signal $x_{k}\left(t\right)$ **Require**: learning rates α, β, λ, window function $w\left(t\right)$ 1: **For** each vibration signal $x_{k}\left(t\right)$ **do** 2:  Compute the STFT for each axial signal with (8) 3:  Perform multi-channel time-frequency fusion with (9) 4: **end for** 5: Randomly initialize parameter θ 6: **while** not done **do**
7:  Sample a batch of tasks $\left\{T_{1},T_{2},T_{3}\right.\cdot \cdot \cdot \left.T_{B}\right\}$ 8:  **for** all $T_{i}$ **do** 9:   Initialize task-specific classifier parameters $\theta_{i,0}=0$ 10:   AEFM feature extraction (channel expansion + SE + spatial attention) 11:   **for** inner loop step j=0  **to** k−1 **do** 12:      Inner-loop classifier update on support set with (16) 13:   **end for** 14:    Computing query set cross-entropy loss with (17) 15:    Computing SupCon loss of task with (19) 16:    Computing RSCML loss of task with (20) 17: **end for** 18: Calculating average cross-entropy loss:
$\;L_{CE^{\prime }}=\frac{1}{B}\sum_{i=1}^{B}\frac{1}{\left|D^{q}\right|}\sum_{\left(x_{q},y_{q}\right)\in D^{q}}L\left(f_{\theta_{i,k}}\left(x_{q}\right),y_{q}\right)$ 19: Calculating average RSCML loss: $\;L_{RSC\mathrm{ML}}=\frac{1}{B}\sum_{i=1}^{B}L_{RSC\mathrm{ML},i}$ 20: Update meta model:
$\;\theta \leftarrow \theta -\beta \nabla_{\theta }L_{RSC\mathrm{ML}}$ 21: **end while**

## 4. Experimental Setup

### 4.1. Data Acquisition and Experimental Scenario

**Figure 8:**
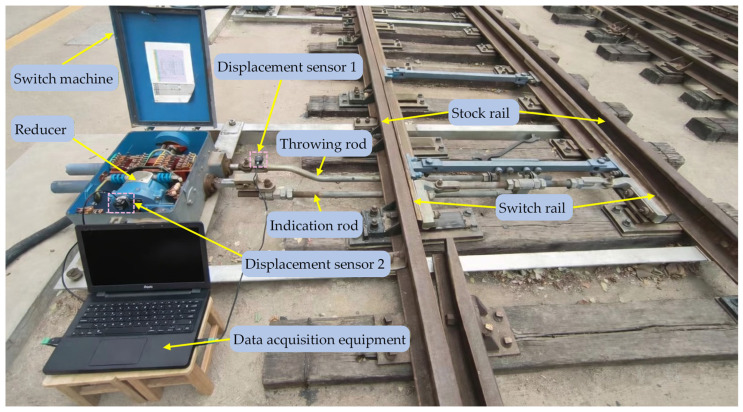
Vibration signals acquisition scenario.

All vibration signals used in this study were acquired at a railway signal training base affiliated with a regional China Railway administration, providing an operationally realistic environment for data collection. During acquisition, two tri-axial vibration sensors (WTVB01-485) were mounted on the throwing rod and the reducer of a ZD6 switch machine to record vibration responses along the X, Y, and Z directions at a sampling rate of 1 kHz, as shown in Figure 8. Based on field experience and the switching duration, an 8 s window was used to capture one complete operation. To improve repeatability and reduce non-fault variability, the same sensor type, measurement points, and sampling configuration were maintained throughout the measurement campaign, and the tri-axial channels data were recorded synchronously.

### 4.2. Dataset Partitioning and Few-Shot Evaluation Setup

In this study, data were collected under six typical operating conditions, including the normal condition and five common fault types. The definitions and sample counts for each condition are listed in Table 1.

For each condition, recordings were acquired at both measurement points, namely the throwing rod and the reducer, resulting in 66 samples per condition at each measurement point. To ensure consistent input length for subsequent analysis, the raw tri-axial signals were standardized in the time domain. Specifically, the first 500 samples of each record were treated as a start-up buffer and set to zero. For each axis, the signal length was formatted to 8000 samples, corresponding to 8 s at 1 kHz. Records shorter than 8000 samples were zero-padded to 8000 samples, whereas longer records were truncated to 8000 samples. As a result, each sample contains three synchronized sequences with a fixed length. To visualize differences among the three vibration axes, we concatenate the tri-axial sequences in temporal order to form a one-dimensional sequence of 24,000 points. This concatenation is used only for data description and is not involved in subsequent model training or evaluation.

**Figure 9:**
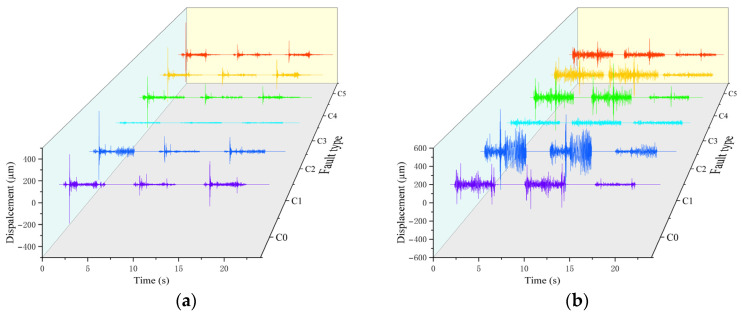
Time domain waveforms of the vibration signals: (**a**) Throwing rod waveforms; (**b**) reducer waveforms.

As shown in Figure 9, under normal operation of the switch machine, the impact responses during both the start-up and locking stages are accompanied by evident changes in vibration displacement. In fault condition C1, an obstruction occurring in the intermediate conversion stage leads to a sustained increase in vibration displacement, while the impact response associated with the locking stage is no longer observable. In fault condition C2, manual adjustment modifies the frictional state of the mechanism, causing the switch machine to enter an idling state due to insufficient driving current. Fault conditions C3 and C4 correspond to obstructions at different traction positions, caused by debris or metal chips, which lead to timing deviations in the impact responses of the throwing rod and a pronounced increase in vibration for the reducer. These two fault types are highly similar. The vibration characteristics of fault condition C5 are most similar to those of the normal condition, and abnormalities appear only near the end of the locking process.

For few-shot evaluation, the dataset was split into training, validation, and test subsets with an approximate ratio of 4:3:3. The split was enforced at the record level, where a record denotes one complete switching operation with vibration signals from both the throwing rod and reducer sensors. To avoid information leakage, all samples from the same record, which includes both sensor locations, were assigned to the same subset, and no record appeared in more than one subset.

In the main experiments, a record-level split was adopted to avoid direct leakage between the throwing rod and reducer signals from the same switching operation. However, potential dependence caused by the acquisition order may still remain. Therefore, an additional conservative partition protocol was further considered. Specifically, for each fault category, the samples were first ordered according to acquisition time and then divided sequentially, with the earliest 40% used for training, the subsequent 30% for validation, and the latest 30% for testing. Compared with the original record-level split, this time-ordered partition is more restrictive because it reduces the residual similarity among temporally adjacent samples. The corresponding results under this protocol are further discussed in Section 5.4.2.

We further assess cross-category generalization to unseen fault types by organizing the data into a source domain and a cross-category test set. In the source domain, training, validation, and in-domain testing used different records from the same fault categories. As shown in Table 2, the source domain contains eight labels by distinguishing throwing rod and reducer vibration signals for conditions C0–C3. Previously unseen fault types were held out from the source domain and reserved for the cross-category test set, which was used only to evaluate cross-category generalization ability.

### 4.3. Implementation Details

For STFT preprocessing, each vibration signal was transformed into a time-frequency spectrogram using a sampling frequency of 1000 Hz, a Hann window, a window length of 256, an overlap length of 128, and an FFT length of 256. The magnitude spectrum was converted to the logarithmic scale in dB and then resized to 128 × 128. Finally, the STFT spectrograms of the three vibration channels were stacked along the channel dimension to form an RGB time-frequency image.

For meta-update optimization, the Adam optimizer is adopted. The outer-loop learning rate β is set to 0.005 to balance convergence speed and training stability, and the inner-loop learning rate α is set to 0.0015 for rapid task adaptation. The weight of the supervised contrastive term λ is set to 0.1, and the L2 regularization coefficient is set to 1 × 10^−5^. The task batch size is set to 4. During training, the model is adapted with five inner-loop gradient steps for each task, and ten inner-loop steps are used during evaluation to ensure sufficient adaptation. Under these settings, all models were trained for 3000 iterations for each experimental configuration. To ensure statistical reliability, we report the mean accuracy and F1-scores over 600 randomly sampled test tasks, together with the corresponding 95% confidence intervals computed across tasks to quantify performance variability. Unless otherwise specified, the random seed was fixed to 1 in all experiments. In addition, representative experiments were repeated under five independent random seeds for robustness analysis, as reported in Section 5.1.3.

## 5. Results and Discussion

To validate the effectiveness and superiority of the proposed RSCML, experiments are conducted on vibration data which we introduced in Section 4, and the results are compared with those of several representative methods. All experiments are implemented in Python 3.12.11 with the PyTorch 2.4.1 framework and executed on a workstation equipped with an Intel Core i7 14700HX CPU (Intel Corporation, Santa Clara, CA, USA) and an NVIDIA GeForce RTX 4070 GPU (NVIDIA Corporation, Santa Clara, CA, USA). And RSCML is compared with a deep learning method, CNN [43], and meta-learning methods including MAML [37], ProtoNet [44], RelaNet [45] and ADMTL [46].

To ensure comparability, all methods were evaluated under the same dataset split and few-shot evaluation setting, while different feature extractors were used according to the experimental settings of each method. For each method, key hyperparameters are selected on the validation split via a limited search within standard ranges reported in prior work; the final settings are summarized in Table 3. The 2D CNN consists of four convolutional blocks, where each block contains a standard CNN stack including a convolution layer, a normalization layer, a ReLU activation function, and a pooling layer.

In the inner loop of meta-learning, we use momentum-free SGD to rapidly update the task-specific parameters. In contrast, the outer loop adopts the Adam optimizer to update the shared initialization parameters. This design preserves the fast adaptation capability of SGD in the inner loop, while taking advantage of Adam in terms of global convergence speed and adaptive learning rate adjustment.

### 5.1. Training Process Analysis

#### 5.1.1. Hyperparameter Sensitivity Analysis

Sensitivity analyses were conducted for two key hyperparameters of the proposed method, namely the weight of the supervised contrastive term λ and the outer-loop learning rate β. Since these two parameters directly affect the balance between supervised contrastive regularization and meta-level optimization, their influence on the final diagnostic performance was further examined under the 3-way 1-shot setting. Specifically, one parameter was varied while the others were kept unchanged, and the corresponding results are shown in Table 4.

As shown in Table 4, the proposed RSCML maintains relatively stable performance within a reasonable range of both the weight of the supervised contrastive term λ and the outer-loop learning rate β. The adopted default settings, namely λ=0.1 and β=0.05, achieve the best results in terms of both accuracy and F1-score. When either parameter deviates from these values, the performance decreases slightly but remains at a relatively high level overall. This indicates that the proposed method has good robustness to these hyperparameters and that the adopted settings are reasonable.

#### 5.1.2. Computational Cost Analysis

In order to evaluate the computational cost of the proposed RSCML under different few-shot settings, training time and test time are further reported. The corresponding results are summarized in Table 5.

As shown in Table 5, the computational cost of the proposed RSCML remains moderate under both the 3-way 1-shot and 5-way 1-shot settings. When the task becomes more challenging, the training time increases from 15.05 min to 21.85 min, and the test time for 600 episodes increases from 19.11 s to 22.49 s. Overall, these results indicate that the proposed method can achieve strong diagnostic performance with an acceptable computational cost under the current experimental setting.

#### 5.1.3. Random Seed Stability Analysis

The stability of the proposed method under different training initializations is further analyzed by repeating RSCML and the strongest competing baseline ADMTL under five independent random seeds in the 3-way 1-shot and 5-way 1-shot settings. The mean accuracy and F1-score, together with their corresponding standard deviations, are reported.

As shown in Table 6 and Table 7, the proposed RSCML demonstrates strong robustness across different random seeds and consistently achieves the best performance under all evaluated settings. In the 3-way 1-shot task, RSCML attains a mean accuracy of 98.92% with a standard deviation of 0.13%, which is clearly higher and more stable than ADMTL (96.16% ± 0.84%). A similar trend can be observed for the F1-score, where RSCML reaches 98.82% ± 0.12%, outperforming ADMTL (95.97% ± 0.90%). Under the more challenging 5-way 1-shot setting, RSCML still maintains the highest mean accuracy and F1-score, achieving 98.70% ± 0.39% and 98.53% ± 0.44%, respectively, whereas ADMTL obtains a 92.37% ± 1.24% accuracy and a 91.84% ± 1.55% F1-score. These results demonstrate that the proposed method not only yields superior average performance but also provides better stability and reliability under different training initializations.

### 5.2. Ablation Experiment

To evaluate the contribution of each component to the overall performance, ablation experiments are conducted on the ZD6 switch machine dataset, examining how the diagnostic accuracy changes as modules are introduced step by step. Four configurations are considered: the basic ANIL framework (ANIL), ANIL with the attention enhanced feature module AEFM (ANIL + AEFM), ANIL with AEFM and SR (ANIL + AEFM + SR), and the complete RSCML method. The classification accuracies of these configurations under the unified 3-way 1-shot and 3-way 5-shot settings are reported in Table 8.

As shown in Table 8, the basic ANIL framework already achieves an accuracy of 96.38% in the 3-way 1-shot setting and 97.53% in the 3-way 5-shot setting, indicating that this strategy can effectively improve the model’s adaptation capability for similar tasks under limited samples. After introducing AEFM, the accuracy further improves to 97.74% and 98.45% under the two settings, respectively, which verifies the effectiveness of the proposed feature extraction module. When stability regularization (SR) is further incorporated, the accuracy increases to 98.64% in the 3-way 1-shot setting and 99.32% in the 3-way 5-shot setting, showing that SR helps reduce overfitting and improves feature stability under episodic training. Finally, by introducing the supervised contrastive term, the complete RSCML achieves the best performance, reaching 99.02% and 99.73% under the 3-way 1-shot and 3-way 5-shot settings, respectively. These results show that the proposed components consistently contribute to performance improvement under both settings.

In both settings, the 95% confidence intervals become progressively narrower as AEFM, SR, and the supervised contrastive term are introduced, indicating improved stability and reliability under episodic evaluation. This trend is particularly clear in the 3-way 5-shot setting, where all methods benefit from more support samples, while the complete RSCML still achieves the highest accuracy together with the narrowest confidence interval. For visual clarity and conciseness, the t-SNE visualization is presented only for the more challenging 3-way 1-shot setting, so as to provide an intuitive illustration of the feature separability achieved by different ablation configurations.

**Figure 10:**
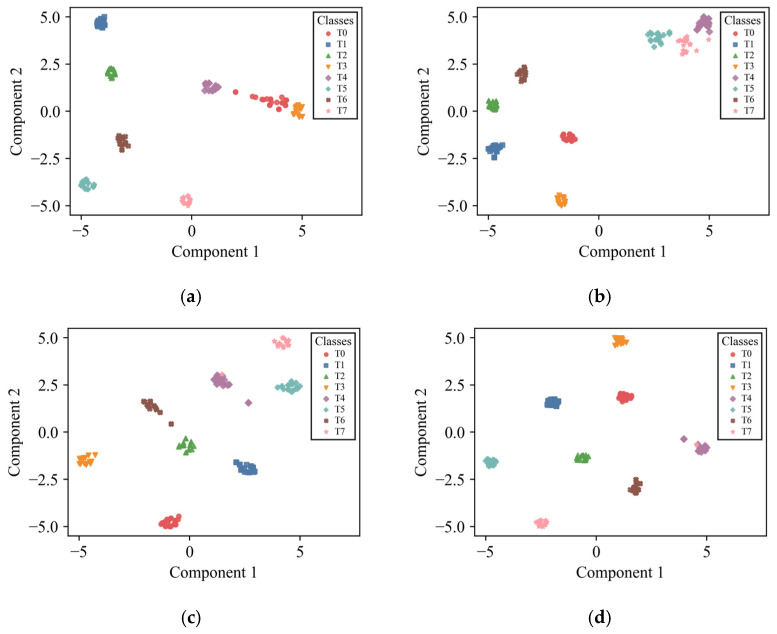
t-SNE visualization for the ablation experiment under the 3-way 1-shot setting: (**a**) ANIL; (**b**) ANIL + AEFM; (**c**) ANIL + AEFM + SR; (**d**) RSCML.

As shown in Figure 10, under the 3-way 1-shot setting, RSCML clearly separated clusters for each fault type, indicating strong discriminative capability in the learned feature space. In contrast, although the other methods achieve reasonably high diagnostic accuracy, the clusters of different fault categories remain relatively close, which increases the likelihood of misclassification. These observations further confirm the effectiveness of the proposed method.

### 5.3. Comparative Experiments

#### 5.3.1. Comparison Under a Unified Backbone

A comparison is further conducted under a unified backbone setting. In the original comparison, the baseline methods used a standard 2D-CNN encoder, whereas RSCML employed the proposed attention-enhanced feature module (AEFM). Since the ablation results show that AEFM improves the basic ANIL framework from a 96.38% to 97.74% accuracy under a 3-way 1-shot setting, an additional unified-backbone analysis was conducted to further separate the contribution of the feature extractor from that of the learning framework. In this analysis, representative meta-learning baseline methods with compatible encoder interfaces were re-implemented using AEFM, while their original learning mechanisms and evaluation settings were kept unchanged. To provide a focused comparison, the experiments were carried out in the 1-shot setting, including both 3-way 1-shot and 5-way 1-shot tasks. Under this setting, the compared methods shared the same feature extractor, such that the remaining performance differences mainly reflected the contribution of the learning framework rather than encoder capacity.

As shown in Table 9, when the backbone is unified as AEFM, the compared meta-learning methods also benefit from the improved feature extractor to different extents. Nevertheless, RSCML still remains the best-performing method in both the 3-way 1-shot and 5-way 1-shot settings. This result suggests that the superiority of RSCML is not solely caused by encoder strength, but is also related to the effectiveness of the proposed learning framework. Therefore, the unified-backbone analysis provides additional evidence supporting the fairness of the comparison.

#### 5.3.2. Comparison with Other Algorithms

The proposed RSCML method is compared with the five baseline methods under the same data partitioning strategy and comparable network depth. Four representative few-shot diagnosis scenarios are constructed, including 3-way 1-shot, 3-way 5-shot, 5-way 1-shot, and 5-way 5-shot. We report both accuracy and F1-score as the evaluation metrics, where accuracy reflects overall correctness and F1-score characterizes the precision and recall balance under few-shot conditions. The accuracy results are summarized in Table 10. And the F1-score results are summarized in Table 11.

**Figure 11:**
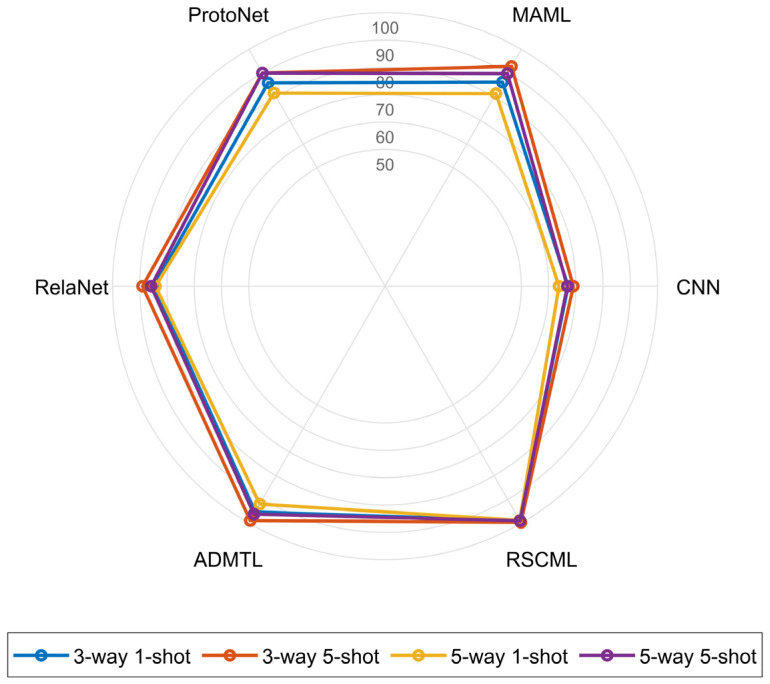
Radar chart of the accuracy achieved by different methods under each setting.

As shown in Figure 11, RSCML achieves accuracies close to or above 99% under all four settings, outperforming the other methods by a clear margin. This result indicates that the proposed method maintains stronger stability and generalization across different task scales and sample regimes. Table 10 further shows that, under the same settings, the conventional CNN shows the weakest results, especially in 1-shot cases where limited supervision prevents learning transferable features, yielding only 67.16% and 63.84% for 3-way 1-shot and 5-way 1-shot. ProtoNet and RelaNet perform better than CNN under scarce labels, suggesting that prototype-based matching improves robustness when samples are limited, but they still degrade notably as task difficulty increases. MAML benefits from meta-training and outperforms CNN, yet it remains behind other meta-learning methods in the most challenging 5-way 1-shot case. ADMTL performs better than the other baselines because it combines attention-guided feature extraction with a meta-transfer training strategy that leverages pre-trained representations and lightweight parameter modulation, enabling more discriminative embeddings and more stable adaptation under scarce samples.

In comparison, the proposed RSCML achieves the best diagnostic performance under all four few-shot settings. Specifically, it attains accuracies of 99.02%, 99.73%, 98.82%, and 99.04% in the 3-way 1-shot, 3-way 5-shot, 5-way 1-shot, and 5-way 5-shot scenarios, respectively, improving upon ADMTL by at least 0.76% to 7.74%. Notably, the associated 95% confidence intervals remain consistently narrow across settings, indicating that RSCML is not only more accurate but also more stable under episodic resampling. In the more challenging 5-way 1-shot setting, RSCML still maintains an accuracy close to 99% under extremely limited samples, further highlighting its effectiveness and robustness.

**Figure 12:**
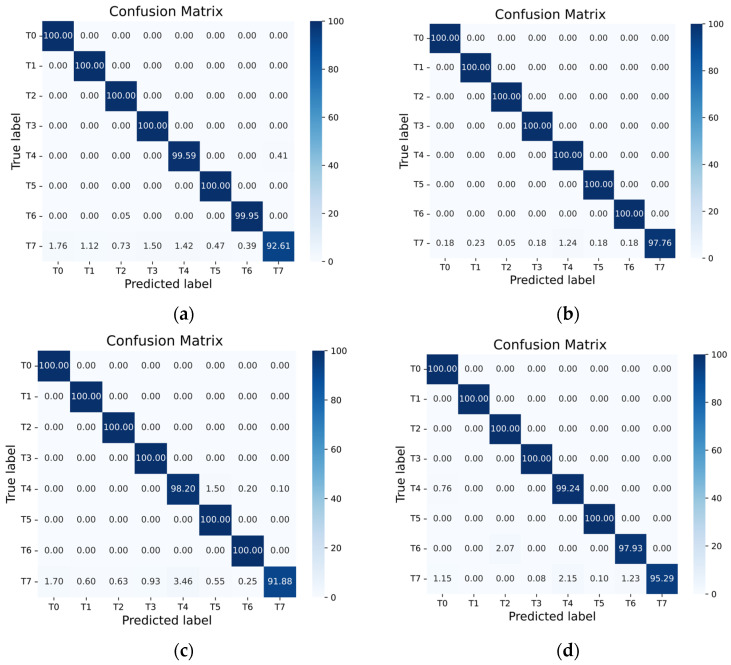
Confusion matrix of RSCML: (**a**) 3-way 1-shot; (**b**) 3-way 5-shot; (**c**) 5-way 1-shot; (**d**) 5-way 5-shot.

To further evaluate the classification performance for each fault category, a confusion matrix is used for analysis. In this study, we obtained the confusion matrix of real fault classes via a label-mapping strategy that converts episode-internal labels back to the original fault labels. As shown in Figure 12, the diagonal entries for most categories are close to 100%, indicating that RSCML achieves high recognition accuracy for the majority of fault types. However, the diagonal value for class T7 is slightly lower than those of the other classes, and a small number of samples are misclassified as T0, T3, and T4. By examining the original vibration signals and their time and frequency characteristics, it can be observed that the T7 fault exhibits similar energy levels and dominant frequency band distributions to these operating conditions over most stages of the switching process. Differences appear only in a brief local action stage, which makes the global time and frequency features of T7 highly close to those of the other categories.

**Figure 13:**
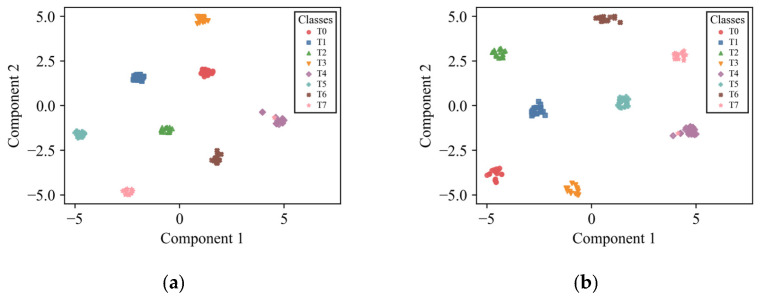

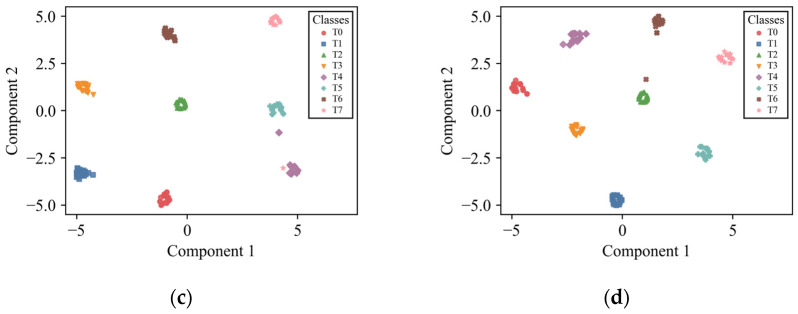
t-SNE visualization of RSCML: (**a**) 3-way 1-shot; (**b**) 3-way 5-shot; (**c**) 5-way 1-shot; (**d**) 5-way 5-shot.

Then t-SNE is applied to visualize the representations from the final layer. As shown in Figure 13, samples from different classes generally form well-separated clusters in the two-dimensional space, with clear inter-class margins and relatively compact intra-class distributions. This observation indicates that the learned features exhibit strong separability. Even in the more challenging 5-way scenario, most clusters remain clearly separated, while only a few classes appear slightly more dispersed. This observation is consistent with the relatively higher misclassification rates of those classes in the confusion matrix.

To comprehensively evaluate the diagnostic performance of the models, we introduce the F1-score as an assessment metric. The F1-score considers both precision and recall, providing a more complete reflection of the model’s diagnostic capability under few-shot conditions. In this study, for each episode, predictions and ground-truth labels are mapped from episode-local indices to the corresponding sampled class identities before computing F1, so that the metric is evaluated under the same episodic label space.

Table 11 compares the F1-score of different methods under four few-shot settings. The proposed RSCML consistently achieves the best F1-score and remains stable as task difficulty increases. ADMTL is the strongest baseline, benefiting from attention-guided meta-transfer learning that improves feature discriminability and supports fast adaptation, but it still lags behind RSCML, particularly in the 5-way 1-shot case. MAML performs markedly better than the conventional CNN due to meta-training for rapid adaptation, yet its F1-score drops in the most challenging low-shot setting, suggesting that gradient-based inner-loop updates can be sensitive to time-frequency information. ProtoNet and RelaNet exhibit moderate robustness by performing distance or relation matching in the feature space, which enables relatively stable performance under few-shot conditions. In contrast, the CNN baseline performs the worst because it relies heavily on sufficient labeled data to learn transferable feature representations.

**Figure 14:**
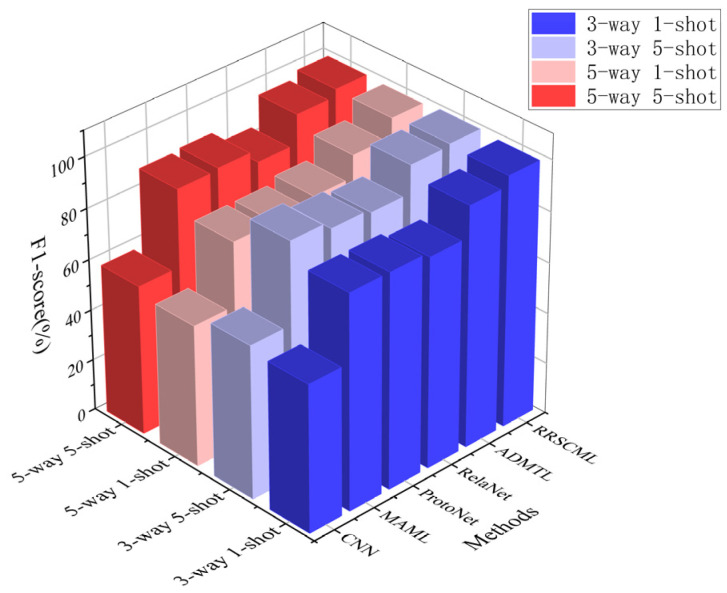
Visualization of F1-scores for different methods.

In contrast, the proposed method achieves the highest F1-scores across all four task settings, reaching 99.72% and 99.03% in the 3-way 5-shot and 5-way 5-shot scenarios, respectively, and maintaining an outstanding 98.65% in the more challenging 5-way 1-shot scenario. Meanwhile, the proposed method also maintains the narrowest 95% confidence interval, which highlights its stability. As shown in Figure 14, the three-dimensional bar chart further illustrates the performance differences of various methods across settings. The proposed method forms the most prominent bar contours in all dimensions, with minimal performance fluctuations across different settings, indicating that it effectively balances high classification accuracy and recall while demonstrating strong stability and robustness.

#### 5.3.3. Statistical Significance Analysis

An additional statistical significance analysis is conducted in the 3-way 1-shot and 5-way 1-shot settings to examine whether the performance advantage of RSCML over the strongest competing baseline ADMTL is statistically significant. Although the two methods were evaluated under the same dataset split and the same few-shot task setting, their episode-level test results were obtained from independently sampled test episodes under their current testing pipelines. Therefore, the Mann–Whitney U test was adopted to compare the episode-level accuracy and F1-score distributions. The corresponding results are reported in Table 12.

The test results show that the proposed RSCML significantly outperforms ADMTL in both settings. In the 3-way 1-shot task, the *p*-values are 2.70 × 10^−36^ for accuracy and 2.90 × 10^−36^ for F1-score. In the 5-way 1-shot task, the *p*-values are 1.09 × 10^−139^ for accuracy and 5.36 × 10^−139^ for F1-score. All these values are far below 0.001, indicating that the observed performance advantage of RSCML is highly statistically significant and is unlikely to be caused by random fluctuations in episode sampling.

### 5.4. Generalization and Robustness Analyses

#### 5.4.1. Cross-Category Generalization Experiment

To evaluate generalization beyond the fault categories observed during meta-training, we conduct a cross-category evaluation where fault categories C4 and C5 are excluded from the source set and used only for testing. The detailed partition of fault labels used in the testing stage is summarized in Table 13. This setting evaluates generalization to unseen fault categories under limited samples, rather than domain adaptation with shared class identities.

**Figure 15:**
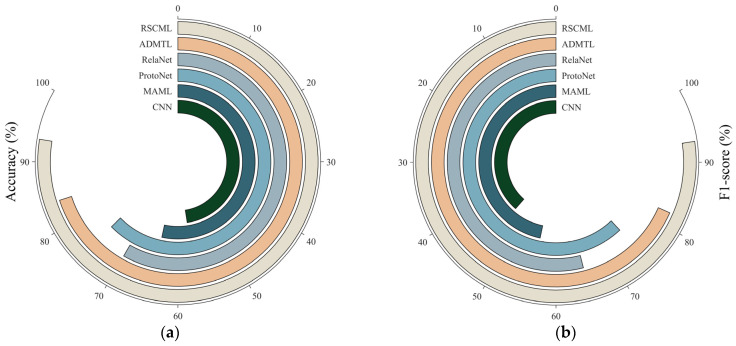
Performance comparison of different methods on the cross-category task: (**a**) Accuracy; (**b**) F1-score.

In the cross-category evaluation, each episode is constructed as a 3-way 5-shot task by randomly sampling three classes from the four unseen categories, and results are averaged over 600 episodes. The experiment results are shown in Table 14 and Figure 15.

This ranking is consistent with how different paradigms handle category shift under scarce labels. The conventional CNN baseline shows the weakest cross-category generalization ability, as it is strongly tied to the source label distribution and lacks robust feature generalization once category shift occurs. MAML can achieve reasonable performance through gradient-based optimization, but it does not fully capture fault-specific features, resulting in limited accuracy when encountering unseen faults. ProtoNet and RelaNet exhibit more stable behavior because distance or similarity matching reduces the need for extensive parameter updates. However, their effectiveness still depends on whether sufficiently discriminative features can be learned from the source categories and generalized to unseen ones. ADMTL further improves stability by combining attention mechanisms and meta-transfer learning, but its class separability and generalization depth remain constrained when inter-class similarity is high.

In contrast, the proposed RSCML consistently demonstrates the most balanced performance across both evaluation metrics, with a notably narrow confidence interval that reflects strong statistical reliability. The advantage of RSCML lies in its ability to enhance fault feature information through multi-channel time-frequency fusion and attention enhancement, and to further strengthen classification performance by means of a regularized supervised contrastive meta-learning structure. As a result, the learned representations remain discriminative even when fault categories differ from those seen during meta-training. This consistency confirms that the proposed method improves not only overall classification accuracy but also class-wise reliability, which is essential for safety-critical fault diagnosis with limited data.

**Figure 16:**
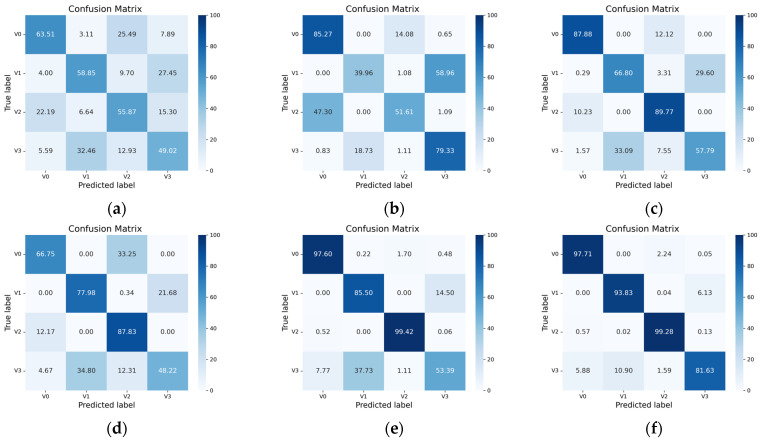
Confusion matrix of cross-category generalization experiment: (**a**) CNN; (**b**) MAML; (**c**) ProtoNet; (**d**) RelaNet; (**e**) ADMTL; (**f**) RSCML.

As shown in Figure 16, the confusion matrix indicates that the proposed RSCML method achieves the highest accuracy across all four fault categories. The misclassification between V1 and V3 is largely mitigated by RSCML, which is difficult for other methods to resolve. In comparison, CNN suffers from notable confusion between several classes, while MAML, ProtoNet, RelaNet, and ADMTL improve performance but still misclassify V1 or V3 because they did not learn transferable feature structures. These results demonstrate that RSCML effectively extracts discriminative representations that generalize well to unseen fault categories.

#### 5.4.2. Robustness Under a More Conservative Split

An additional evaluation is conducted under the more conservative time-ordered split described in Section 4.2. Compared with the original record-level split, this setting reduces the similarity between temporally adjacent samples and makes the evaluation more challenging. The corresponding results are presented in Table 15.

As shown in Table 15, introducing the more conservative time-ordered partition reduces the potential dependence among samples and makes the evaluation more challenging. Under this stricter protocol, ADMTL suffers from a relatively obvious performance degradation, especially in the 5-way 1-shot setting. By comparison, the proposed RSCML shows only marginal changes in both accuracy and F1-score under the same partition. Moreover, RSCML still remains the best-performing method in all evaluated settings. This demonstrates that the effectiveness of the proposed framework is not strongly influenced by the adopted split strategy and further supports its robustness under a less optimistic and more conservative data partition.

## 6. Conclusions

This study addresses switch machine fault diagnosis in field environments where labeled fault samples are difficult to obtain and data scarcity is inherent. We develop a regularized supervised contrastive meta-learning (RSCML) method that integrates multi-sensor, multi-channel time-frequency fusion with attention enhancement to achieve reliable fault diagnosis under limited on-site labeled data. First, the MCTF module converts tri-axial vibration signals into three-channel time-frequency images to preserve complementary multi-axis information in a unified representation and facilitate subsequent cross-channel feature learning. Next, an attention-enhanced feature extraction module (AEFM) is developed by integrating channel expansion with SE channel attention and spatial attention to highlight fault-sensitive time-frequency patterns under limited samples. Finally, a regularized supervised contrastive ANIL framework is introduced. It combines multi-loss learning with stability regularization (SR) to suppress overfitting and to promote more discriminative embeddings by tightening intra-class clustering and enlarging inter-class margins. This design supports reliable diagnosis in both few-shot and cross-category settings. Ablation results confirm the effectiveness of each module, and experiments on the ZD6 switch machine dataset show that RSCML consistently outperforms competing methods across multiple N-way and K-shot tasks, with clear advantages also observed in cross-category generalization to unseen fault categories within the current dataset setting.

In the present study, effective fault diagnosis has been achieved for a dataset collected from a single switch machine under relatively focused fault categories and operating conditions. Future work will incorporate additional modalities such as current and power signals, explore more explicit modeling of inter-axis correlations, and extend the framework to multi-device and multi-condition fault diagnosis through the integration of transfer learning and meta-learning.

## Figures and Tables

**Table 1:** Operating conditions and fault descriptions of the switch machine.

Conditions	Descriptions	Samples
C0	Normal condition	66
C1	Incomplete-locking power-off fault	66
C2	No load condition	66
C3	Foreign matter stuck on switch rail 1	66
C4	Foreign matter stuck on switch rail 2	66
C5	Locking failure fault	66

**Table 2:** Fault labels in the source domain.

Conditions	Parts	Labels	Parts	Labels
C0	Throwing rod	T0	Reducer	T4
C1	Throwing rod	T1	Reducer	T5
C2	Throwing rod	T2	Reducer	T6
C3	Throwing rod	T3	Reducer	T7

**Table 3:** Structural description of the compared methods.

Methods	Feature Extractor	Classifier	Optimizer
CNN	2D-CNN	Linear, Softmax	Standard Adam
MAML	2D-CNN	Linear, Softmax	SGD + Adam
ProtoNet	2D-CNN	Metric function	Standard Adam
RelaNet	2D-CNN	Metric function	Standard Adam
ADMTL	2D-CNN	Linear, Softmax	Standard Adam
RSCML	AEFM	Linear, Softmax	SGD + Adam

**Table 4:** Hyperparameter sensitivity analysis of the proposed RSCML.

Hyperparameter	Value	Accuracy	F1-Score
λ	0.01	98.45%	98.30%
λ	0.1	99.02%	98.87%
λ	0.2	98.77%	98.64%
β	0.003	98.87%	98.82%
β	0.005	99.02%	98.87%
β	0.007	98.51%	97.96%

**Table 5:** Computational cost of the proposed RSCML under different few-shot settings.

Setting	Training Time (min)	Test Time (s)	Number of Test Episodes
3-way 1-shot	15.05	19.11	600
5-way 1-shot	21.85	22.49	600

**Table 6:** Mean accuracy with standard deviations across five random seeds.

Methods	Setting	Seed					Mean ± (Std)
		1	2	3	4	5	
ADMTL	3-way 1-shot	95.31%	96.73%	95.00%	97.07%	96.71%	96.16 ± 0.84%
	5-way 1-shot	91.99%	93.92%	90.24%	92.60%	93.09%	92.37 ± 1.24%
RSCML	3-way 1-shot	99.02%	98.68%	98.89%	98.93%	99.07%	98.92 ± 0.13%
	5-way 1-shot	98.82%	97.93%	98.94%	98.83%	98.97%	98.70 ± 0.39%

**Table 7:** Mean F1-score with standard deviations across five random seeds.

Methods	Setting	Seed					Mean ± (Std)
		1	2	3	4	5	
ADMTL	3-way 1-shot	94.94%	96.65%	94.81%	96.88%	96.58%	95.97 ± 0.90%
	5-way 1-shot	91.49%	93.77%	89.15%	92.00%	92.77%	91.84 ± 1.55%
RSCML	3-way 1-shot	98.87%	98.59%	98.81%	98.86%	98.95%	98.82 ± 0.12%
	5-way 1-shot	98.65%	97.65%	98.78%	98.74%	98.81%	98.53 ± 0.44%

**Table 8:** Accuracy of RSCML ablation experiment.

Methods	3-Way 1-Shot	3-Way 5-Shot
ANIL	96.38 ± 0.61%	97.53 ± 0.38%
ANIL + AEFM	97.74 ± 0.43%	98.45 ± 0.22%
ANIL + AEFM + SR	98.64 ± 0.36%	99.32 ± 0.11%
RSCML	99.02 ± 0.33%	99.73 ± 0.07%

**Table 9:** Comparison under a unified AEFM backbone.

Methods	3-Way 1-Shot		5-Way 1-Shot	
	Accuracy	F1-Score	Accuracy	F1-Score
ProtoNet	92.03 ± 0.55%	91.57 ± 0.60%	85.29 ± 1.03%	84.22 ± 1.15%
RelaNet	90.19 ± 1.31%	89.49 ± 1.46%	86.33 ± 1.01%	85.36 ± 1.12%
RSCML	99.02 ± 0.33%	98.87 ± 0.43%	98.82 ± 0.31%	98.65 ± 0.39%

**Table 10:** Fault diagnosis accuracy of different methods.

Methods	3-Way 1-Shot	3-Way 5-Shot	5-Way 1-Shot	5-Way 5-Shot
CNN	67.16 ± 1.24%	69.10 ± 1.23%	63.84 ± 0.97%	66.90 ± 0.89%
MAML	86.18 ± 1.13%	92.82 ± 0.92%	81.36 ± 0.91%	89.80 ± 0.72%
ProtoNet	85.87 ± 0.69%	90.02 ± 0.95%	81.59 ± 1.14%	90.01 ± 0.49%
RelaNet	84.88 ± 1.90%	89.01 ± 1.44%	84.22 ± 1.17%	85.87 ± 1.02%
ADMTL	95.31 ± 0.64%	98.97 ± 0.23%	91.99 ± 0.57%	96.23 ± 0.24%
RSCML	99.02 ± 0.33%	99.73 ± 0.07%	98.82 ± 0.31%	99.04 ± 0.09%

**Table 11:** F1-score of different methods.

Methods	3-Way 1-Shot	3-Way 5-Shot	5-Way 1-Shot	5-Way 5-Shot
CNN	58.34 ± 1.25%	60.50 ± 1.55%	55.62 ± 1.11%	58.80 ± 0.89%
MAML	85.02 ± 1.25%	92.59 ± 0.95%	80.38 ± 0.96%	89.05 ± 0.79%
ProtoNet	84.96 ± 0.75%	89.59 ± 1.01%	80.41 ± 1.24%	89.79 ± 0.51%
RelaNet	82.94 ± 2.19%	88.17 ± 1.61%	82.89 ± 1.29%	84.81 ± 1.14%
ADMTL	94.94 ± 0.74%	98.94 ± 0.26%	91.49 ± 0.65%	96.20 ± 0.24%
RSCML	98.87 ± 0.43%	99.72 ± 0.07%	98.65 ± 0.39%	99.03 ± 0.09%

**Table 12:** Statistical significance analysis between RSCML and ADMTL.

Setting	Measure	RSCML	ADMTL	*p*-Value
3-way 1-shot	Accuracy	99.02 ± 0.33%	95.31 ± 0.64%	2.70 × 10^−36^
3-way 1-shot	F1-score	98.87 ± 0.43%	94.94 ± 0.74%	2.90 × 10^−36^
5-way 1-shot	Accuracy	98.82 ± 0.31%	91.99 ± 0.57%	1.09 × 10^−139^
5-way 1-shot	F1-score	98.65 ± 0.39%	91.49 ± 0.65%	5.36 × 10^−139^

**Table 13:** Fault labels in the cross-category test set.

Conditions	Parts	Labels	Parts	Labels
C4	Throwing rod	V0	Reducer	V1
C5	Throwing rod	V2	Reducer	V3

**Table 14:** Fault diagnosis accuracy and F1-score in cross-category generalization experiment.

Methods	Accuracy	F1-Score
CNN	57.11 ± 0.97%	46.30 ± 1.06%
MAML	64.01 ± 1.14%	56.05 ± 1.32%
ProtoNet	75.22 ± 0.93%	74.45 ± 0.99%
RelaNet	69.98 ± 1.03%	64.87 ± 1.31%
ADMTL	83.99 ± 0.49%	82.03 ± 0.49%
RSCML	93.08 ± 0.54%	92.84 ± 0.57%

**Table 15:** Performance comparison under the original and more conservative data split.

Methods	Setting	Original Split Accuracy	Original Split F1-Score	Conservative Split Accuracy	Conservative Split F1-Score
ADMTL	3-way 1-shot	95.31 ± 0.64%	94.94 ± 0.74%	92.67 ± 0.70%	92.19 ± 0.77%
	5-way 1-shot	91.99 ± 0.57%	91.49 ± 0.65%	89.52 ± 0.81%	87.87 ± 0.98%
RSCML	3-way 1-shot	99.02 ± 0.33%	98.87 ± 0.43%	98.94 ± 0.39%	98.86 ± 0.43%
	5-way 1-shot	98.82 ± 0.31%	98.65 ± 0.39%	98.65 ± 0.36%	98.51 ± 0.42%

## Data Availability

Due to privacy restrictions, the data provided in this study are available on request from the corresponding author.
